# Clinical Reasoning: A 75-Year-Old Man With Dementia, Incontinence, and Gait Dysfunction

**DOI:** 10.7759/cureus.9311

**Published:** 2020-07-21

**Authors:** Kent R Richter, Ryan Naylor, Jeremy K Cutsforth-Gregory, Benjamin D Elder

**Affiliations:** 1 Neurosurgery, Mayo Clinic Alix School of Medicine, Mayo Clinic, Phoenix, USA; 2 Neurosurgery, Mayo Clinic, Rochester, USA; 3 Neurology, Mayo Clinic, Rochester, USA

**Keywords:** idiopathic normal pressure hydrocephalus, cervical stenosis, cervical spondylotic myelopathy

## Abstract

Idiopathic normal pressure hydrocephalus (iNPH) is a progressive neurological disorder characterized by gait apraxia, cognitive decline, and urinary incontinence. It can be difficult to diagnose iNPH as the symptoms may overlap with other neurodegenerative diseases including ​cervical spondylotic myelopathy. Cervical spondylotic myelopathy is a progressive degenerative disease in which compression of the cervical spinal cord causes gait disturbances and imbalance, loss of dexterity and strength in the hands, and, at late stages, urinary dysfunction. As with iNPH, increased age is associated with higher incidence and prevalence. Surgical decompression of the cervical spinal cord is the treatment of choice in patients with progressive myelopathy. Accordingly, iNPH and cervical myelopathy may both present with progressive gait impairment and incontinence, especially in the elderly.

The case presented here demonstrates that both iNPH and cervical myelopathy may present simultaneously and result in gait disturbances and imbalance in some patients. For patients with suspected iNPH and myelopathic findings on examination, it is prudent to obtain a cervical spine MRI to assess for cervical stenosis. Moreover, cervical stenosis can mask the effect of cerebrospinal fluid diversion in patients with comorbid iNPH and cervical myelopathy. Therefore, the differential for patients who have symptomology suggestive of iNPH should include cervical spine myelopathy, with considerations for possible cervical decompression in addition to placement of a ventriculoperitoneal shunt.

## Introduction

The case presented here demonstrates that both idiopathic normal pressure hydrocephalus (iNPH) and cervical myelopathy may present simultaneously and result in gait disturbances and imbalance in some patients. For patients with suspected iNPH and myelopathic findings on examination, a cervical spine MRI should be considered to assess for cervical stenosis. Cervical stenosis can mask the effect of CSF diversion in patients with comorbid iNPH and cervical myelopathy.

## Case presentation

A 75-year-old man presented with progressive deterioration in gait and balance over a two-year period. One year after symptom onset, he was diagnosed with Parkinson’s disease and was started on carbidopa/levodopa but enjoyed no clinical improvement. Despite the regular use of a walker, he sustained several falls in the month, leading up to his initial evaluation at our institution. He also developed urinary urge incontinence and mild cognitive impairment over a three- to six-month period. The patient denied loss of dexterity or Lhermitte phenomenon. On examination, he scored 29/38 on the Kokmen Short Test of Mental Status, losing points on registration, recall, calculation, and construction. His gait was moderately slow and shuffling, with increased number of steps to turn, reduced arm swing, forward-leaning posture, and Trendelenburg sign. He had full strength in the bilateral upper and lower extremities and no sensory abnormalities. He had symmetric moderate hyperreflexia of the biceps, brachioradialis, and quadriceps, and had moderate spasticity in both lower extremities. There was no Hoffman sign present, and he had mute plantar responses bilaterally.

**Figure 1:**
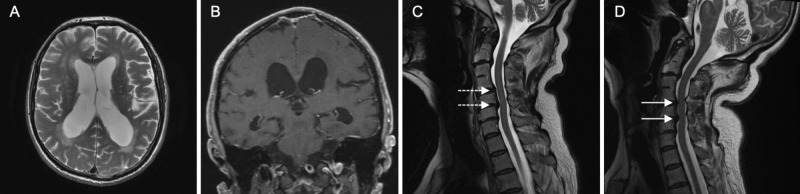
Patient’s Brain and Cervical Spine MRI (A) Axial T2-weighted brain MRI demonstrating mild ventriculomegaly with Evan’s index 0.32. (B) Coronal T1-weighted contrast-enhanced brain MRI demonstrating enlargement of the temporal horns, tight-high convexity and medial surface subarachnoid spaces, enlarged left Sylvian fissure, and a callosal angle measuring 73.9°. (C) Sagittal T2-weighted cervical spine MRI; dotted arrows indicate mild stenosis at C4-C5 and moderate stenosis at C5-C6 with the patient’s head in the neutral position. (D) Sagittal T2-weighted cervical spine MRI; solid arrow depicts severe stenosis at C4-C5 and C5-C6 in extension.

MRI of the brain (Figures 1A, 1B) demonstrated moderate ventriculomegaly with an Evans index of 0.32, tight high-convexity and medial surface subarachnoid spaces, a modestly enlarged left Sylvian fissure, and a callosal angle of 73.9˚, consistent with the disproportionately enlarged subarachnoid space hydrocephalus (DESH) variant of iNPH. MRI of the cervical spine (Figure 1C) demonstrated moderate central canal stenosis at C5-C6. Given the high index of suspicion for iNPH, the patient underwent a high-volume lumbar puncture, which revealed a normal opening pressure of 132 mmH_2_O. Prior to withdrawal of 30-mL cerebrospinal fluid (CSF), the patient took an average of 20.4 seconds to walk 10 meters (standard deviation [SD]: 2.6 seconds; n = 4) and an average of 5.8 steps for every 180˚ turn. After CSF removal, he walked 10 meters in an average of 17.6 seconds (SD: 1.4 seconds; n = 4) and, again, took an average of 5.8 steps per 180˚ turn. There was no statistically significant difference in the time it took the patient to walk 10 meters before and after high-volume lumbar tap (p = 0.10) or in the number of steps to turn.

The differential diagnosis of progressive gait apraxia, mild cognitive impairment, and urge incontinence includes iNPH, cervical spondylotic myelopathy, typical and atypical parkinsonism, hip osteoarthritis, and combinations of these. As described previously, the brain MRI was consistent with iNPH, but the patient had an equivocal response to the high-volume lumbar puncture. The cervical spine MRI revealed diffuse degenerative changes with moderate stenosis at C5-C6, which, in combination with the upper motor neuron findings on examination (even without weakness or loss of dexterity in the hands), suggested cervical myelopathy. The patient had already been treated with carbidopa/levodopa, but without any benefit. Finally, plain radiographs revealed advanced osteoarthritis of the hips and sacroiliac joints, but this diagnosis did not explain the patient’s cognitive impairment or urinary symptoms. Based on the underwhelming response to high-volume CSF removal and mobile spondylolisthesis in the cervical spine, a flexion-extension MRI of the cervical spine was pursued, which demonstrated slight progression of the canal effacement at C5-C6 in extension but, surprisingly, also demonstrated dynamic compression of the spinal cord at C4-C5 (Figure 1D).

Anterior cervical discectomy and fusion (ACDF) at C4-C5 and C5-C6 was performed, without complication, to decompress the cervical spinal cord. He continued to suffer progressive deterioration of his gait and balance over the ensuing year, however, and therefore returned for repeat lumbar tap test. Prior to this second high-volume lumbar puncture, the patient took 31.1 seconds on average to walk 10 meters (SD: 3.6 seconds; n = 4) and an average of 7.6 steps per 180˚ turn. The opening pressure of the lumbar puncture was 194 mmH_2_O. After removing 30-mL CSF, it took the patient an average of 16.0 seconds to walk 10 meters (SD: 0.8 seconds; n = 4) and 5.0 steps to turn 180˚. The difference in time to complete a 10-meter walk (Video 1) before and after lumbar puncture was statistically significant (p < 0.01), as was the number of steps per 180˚ turn (p < 0.01), both determined using a two-tailed Student’s t-test. Based on this clear response to temporary CSF removal, the patient underwent placement of a right parietal ventriculoperitoneal shunt. Three months after shunting, the patient was no longer using a walker and just used a cane when outside the house and had not fallen.

**Video 1:** : Patient’s 10-Meter Walk Test

## Discussion

INPH is a progressive neurological disorder characterized by gait apraxia, cognitive decline, and urinary incontinence [1]. Neuroimaging demonstrates ventriculomegaly despite normal lumbar puncture opening pressure [2]. The incidence of iNPH is 0.5 to 5.5 per 100,000 people per year and increases as a function of age [3,4]. It can be difficult to diagnose iNPH as the symptoms may overlap with other neurodegenerative diseases [5]. Treatment for iNPH is prolonged CSF diversion through shunting, which is most effective in ameliorating gait dysfunction, although as many as 20% of shunted patients fail to achieve full resolution of gait abnormalities [6]. The decision as to whether a patient is a good candidate for shunting can be determined by their response to high-volume lumbar tap [7].

Cervical spondylotic myelopathy is a progressive degenerative disease in which compression of the cervical spinal cord causes gait disturbances and imbalance, loss of dexterity and strength in the hands, and, at late stages, urinary dysfunction [6,8]. As with iNPH, increased age is associated with higher incidence and prevalence. Surgical decompression of the cervical spinal cord is the treatment of choice in patients with progressive myelopathy [9]. Accordingly, iNPH and cervical myelopathy may both present with progressive gait impairment and incontinence, especially in the elderly [10].

The case presented here demonstrates that both iNPH and cervical myelopathy may present simultaneously and result in gait disturbances and imbalance in some patients. For patients with suspected iNPH and myelopathic findings on examination, it is prudent to obtain a cervical spine MRI to assess for cervical stenosis. Moreover, cervical stenosis can mask the effect of CSF diversion in patients with comorbid iNPH and cervical myelopathy. This phenomenon may be, in part, why up to 20% of patients with iNPH fail to achieve full gait resolution after shunting. Therefore, the differential for patients who have symptomology suggestive of iNPH should include cervical spine myelopathy, with considerations for possible cervical decompression in addition to placement of a ventriculoperitoneal shunt.

## Conclusions

For patients with suspected iNPH and myelopathic findings on examination, it is prudent to obtain a cervical spine MRI to assess for cervical stenosis. The differential for patients who have symptomology suggestive of iNPH should include cervical spine myelopathy, with considerations for possible cervical decompression in addition to placement of a ventriculoperitoneal shunt.
